# Milking dynamics following individual quarter dry-off in Holstein cows in an automatic milking system

**DOI:** 10.3168/jdsc.2025-0878

**Published:** 2025-12-05

**Authors:** Clara Ibarguren, Jason Lombard, Juan Velez, Constanza Hernandez-Gotelli, Pablo Pinedo

**Affiliations:** 1 Department of Animal Sciences, Colorado State University, Fort Collins, CO 80523; 2 Department of Clinical Sciences, College of Veterinary Medicine and Biomedical Sciences; AgNext, Colorado State University, Fort Collins, CO 80523; 3 Aurora Organic Farms, Platteville, CO 80651

## Abstract

Individual quarter dry-off (QDO) is a targeted management strategy used to address persistent IMI that lead to chronic subclinical mastitis, as well as cases of clinical mastitis that are recurrent or unresponsive to treatment. Although the interest in the use of individual QDO as a non-antimicrobial strategy for mastitis control is growing, the impact of this management on subsequent milk production has not been widely explored. Moreover, detailed information on the individual performance of the remaining functional quarters following QDO is scarce. The objective of this observational study was to investigate the effect of lactational QDO following clinical mastitis on short-term milk yield in the remaining individual quarters. This retrospective study was conducted in a commercial dairy farm located in northeast Colorado, equipped with an automatic milking system. The analysis included 114 multiparous cows with one quarter dried off through abrupt cessation of milking following unresponsive clinical mastitis therapy. For comparison, one healthy control cow was matched to each affected cow based on DIM and parity number. Individual quarter milk yield of the remaining functional quarters and from control cows was collected for each milking visit from the on-farm management software and summed as a daily value per quarter for the 30 d following QDO. The herd average DIM at the peak of lactation (68 DIM) was considered to categorize the study cows based on their DIM at QDO into prepeak and postpeak groups. All the analyses were conducted separately for these 2 stage of lactation groups and cows were also categorized based on their dry quarter location (DQL). Least squares means (SE) for daily average milk yield per functional quarter and per cow up to 30 d post QDO were calculated and compared among DQL groups (including matched control cows) using ANOVA for repeated measures analysis, with cow ID as the repeated statement, with compound symmetry selected as the covariance structure. Multivariable models included DQL as explanatory variable of interest and DIM at QDO and calving season as potential covariates. In addition, milk yield curves up to 30 d post-QDO were built for milk yield per DQL using daily LSM calculated by repeated measures analysis. Cow-level milk yield was also compared between DQL groups, including unaffected control cows using *t*-test for repeated measures analysis. Differences in quarter milk yield were only identified for the prepeak group (≤68 DIM). Milk yield from the left rear and right rear quarters was smaller in control cows than in cows with the right front quarter dried off. When total milk yield per cow was compared within the prepeak group, control cows had greater yield than cows subjected to QDO of the right rear quarter. In the postpeak group, control cows had the highest milk yield compared with the 4 groups of cows with a dry quarter. In conclusion, following QDO, the levels of milk yield compensation in the remaining functional quarters were variable and smaller when QDO occurred post peak (>68 DIM). Nonetheless, in most cases the cow-level milk yield remained lower in 3-quarter cows compared with unaffected controls.

## Highlights

• Individual QDO is a targeted strategy to address persistent intramammary infections that lead to chronic subclinical mastitis.
• We investigated the effect of lactational QDO following clinical mastitis on short-term milk yield in the remaining individual quarters.
• Following QDO, milk yield compensation in the remaining functional quarters was variable and smaller when QDO occurred postpeak.
• In most cases, cow-level milk yield remained lower in 3-quarter cows compared with unaffected controls.

## Graphical Abstract

**Figure:**
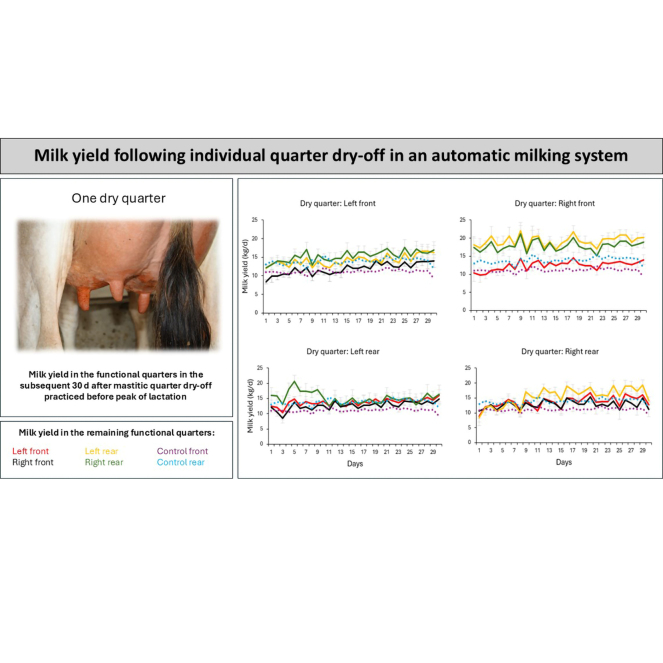
**Summary:** An alternative to conventional therapeutic methods for managing chronic subclinical mastitis and recurrent or unresponsive clinical cases is the individual quarter dry-off (QDO) procedure, which involves ceasing milking from the affected quarter to control infection. While QDO is practiced in some regions, particularly among organic producers, its broader adoption is limited by a lack of data on the subsequent performance of treated cows. This study aimed to evaluate the effect of lactational QDO following clinical mastitis on short-term milk yield in the remaining quarters. Our findings indicate that milk yield compensation in the remaining functional quarters after QDO was variable and generally smaller when QDO occurred after peak lactation. Overall, cow level milk yield remained lower in 3-quarter cows compared with unaffected controls.

Despite important advances in the control of mastitis, this condition remains prevalent in dairy cows, leading to decreased milk yield and quality, compromised animal welfare, and substantial economic losses (Rajala-Schultz et al., 1999; Ruegg, 2018). Current treatment protocols for clinical mastitis emphasize targeted antibiotic use, particularly for gram-positive infections, while allowing spontaneous resolution in other cases (Ruegg, 2018). In contrast, the treatment of subclinical mastitis is often avoided due to the high costs associated with therapy and milk withdrawal, as well as increasing regulatory and consumer pressure to reduce antibiotic usage (Francoz et al., 2017). Nonetheless, mastitis prevention and treatment account for the majority of antimicrobials administered to adult dairy cows (Pol and Ruegg, 2007; Stevens et al., 2016). In response, there are growing efforts exploring novel treatment options for the control of mastitis (Ruegg, 2018).

An alternative to conventional therapeutic methods for managing chronic subclinical mastitis and unresponsive or recurrent clinical cases is the individual quarter dry-off (**QDO**) procedure, which involves the cessation of milking from the affected quarter to control infection (Middleton and Fox, 2001; Skarbye et al., 2018; Wieland and Skarbye, 2024).

Quarter dry-off may be performed by infusing antiseptics such as chlorhexidine or povidone-iodine into the affected quarter, or without chemical intervention by excising the teat through banding, or simply by ceasing to milk the affected quarter (Erskine et al., 2003; Vaarst et al., 2006). As reported by Skarbye et al. (2018), milking interruption can be implemented either through abrupt cessation (i.e., abrupt QDO) or by milking the quarter once or twice during the week following the initiation of QDO (i.e., gradual QDO).

Following the cessation of milking, intramammary pressure and tissue firmness increase, initiating active glandular involution through apoptotic mechanisms, reducing milk production and secretion (Blau et al., 2019). Skarbye et al. (2018) reported that between d 7 and 14 after milking cessation, QDO was associated with quarter swelling, presumably due to distension caused by milk accumulation in the affected quarter. By d 40, production had declined enough to relieve distension, closely mirroring routine drying off.

The main goals of QDO are to eliminate the infection by causing fibrosis of the affected quarter, thus reducing the risk of further pathogenic change or systemic effects on the cow, as well as to avoid penalties associated with high bulk tank SCC and to limit the spread of infections (Erskine et al., 2003; Leitner et al., 2012; USDA, 2016). As reviewed by Erskine et al. (2003), the QDO procedure may have some benefit in genetically superior animals within a herd or for cows that are to be maintained until calving. In addition, QDO has been reported as an effective strategy for controlling persistent contagious pathogens, such as *Staphylococcus aureus*, by eliminating the affected mammary quarter as a reservoir of infection. In a technical note, Middleton and Fox (2001) compared the efficacy of quarter infusion with chlorhexidine (1 g in a 28-mL base, administered after 2 milkings 24 h apart) and povidone-iodine (120 mL of a 5% solution [0.5% iodine]) in inducing lactation cessation in *S. aureus*-infected quarters. In both treatment groups, the quarters were not milked for the remainder of the lactation. Notably, povidone-iodine was more effective, resulting in permanent cessation of lactation in all treated quarters. In contrast, 71% of the chlorhexidine-treated quarters resumed lactation in the subsequent cycle.

In consequence, the implementation of QDO procedure represents a valid strategy for specific mastitis cases and could be especially relevant in organic dairy systems, where statutory restrictions limit or prohibit antibiotic use (Vaarst et al., 2006). Nonetheless, as presented by Wieland and Skarbye (2024), the selection of cows and quarters should be made considering factors such as the productive life of the cow, the infectious status of the other quarters, and the predicted future milk production.

However, while QDO is practiced in certain regions, and particularly among organic producers (Vaarst et al., 2006), its broader adoption remains limited by lack of data on the subsequent performance of treated cows. Although some studies suggest that remaining functional quarters may compensate for the production loss, potentially minimizing the overall impact on milk yield (Hamann and Reichmuth, 1990), detailed information regarding post QDO production at the quarter level is missing.

With the increasing adoption of automatic milking systems (**AMS**), where records for individual quarter yield are produced for each milking, there is potential to examine more in detail how QDO affects the milking dynamics of the remaining individual quarters. We hypothesized that, following QDO, the remaining functional quarters would compensate by increasing their milk yield, resulting in overall cow yields comparable to those of unaffected controls. The objective of this study was to investigate the effect of lactational QDO following clinical mastitis on short-term milk yield in the remaining individual quarters.

All research procedures were approved by the Colorado State University IACUC Waiver Subcommittee (protocol #4907). This retrospective observational study was conducted on a commercial dairy farm in northeast Colorado, housing 3,700 lactating cows in freestall barns. Cows were milked using an AMS consisting of 62 voluntary milking system units (DeLaval International AB, Tumba, Sweden) operating within a guided-flow configuration (milking-first).

Cows calved in a designated maternity pen, where they remained for 24 to 48 h before being moved to a fresh pen equipped with freestalls until 18 to 20 DIM. Subsequently, cows were transferred to the lactation pen, where both primiparous and multiparous cows were milked using an AMS.

The average stocking density during the study was 62 ± 5 cows per AMS unit. Cows had ad libitum access to clean water and were fed a TMR twice daily, formulated according to NRC (2001) guidelines. Farm personnel monitored cow activity using the DeLaval Activity Meter System in conjunction with the DelPro Farm Management system (DeLaval International AB, Tumba, Sweden) for reproductive and health management purposes.

Milk yield per quarter was measured by 4 individual milk flow meters in each of the voluntary milking units and transmitted for storage into DelPro Farm Management software version 4.x for each milking visit. Cow information, including calving date, parity number, and health events, were retrieved from the on-farm management software DairyComp 305 (XE software, version 24.10.1066, Valley Ag Software, Tulare, CA). Data were organized by cow's unique ID number and lactation number. The final dataset included cow ID, calving date, calving season (4 meteorological seasons), lactation number, date and time of each milking event, individual quarter and total milk yield per AMS visit, and the corresponding dry quarter location (**DQL**).

Mastitis diagnosis was performed by farm personnel in accordance with established herd health protocols. Daily alerts generated by the AMS based on the DeLaval Mastitis Detection Index that considers milk electrical conductivity, presence of blood in milk, and milking interval, along with reductions in general activity monitored via activity collars, were reviewed. Cows signaled by these systems were sorted for clinical examination. A diagnosis of mastitis was confirmed if clinical signs of udder inflammation or visibly abnormal milk secretion were observed in one or more quarters (Kelton et al., 1998).

After identifying cows with clinical mastitis, farm staff aseptically collected milk samples from the affected quarters for bacteriological analysis using tri-plates from the AccuMast On-Farm Mastitis Culture Test Kit (FERA Diagnostics and Biologicals, Ithaca, NY). Treatment decisions were guided by the culture results, following the farm's established protocol. Briefly, cows identified with gram-positive infections received intramammary antimicrobial therapy (amoxicillin intramammary suspension). Depending on severity, gram-negative cases also received intramammary antimicrobial therapy (ceftiofur intramammary suspension), combined with systemic support therapy. Cows with milk cultures identifying *Staphylococcus aureus* were immediately transferred to a separate dairy with conventional milking practices. Cows with no bacterial growth (culture-negative) results were re-evaluated clinically, and treatment decisions were based on the presence and severity of clinical signs (i.e., localized signs in the affected quarter or systemic signs, or both).

If the prescribed treatment required a milk withdrawal period, affected cows were relocated to the hospital pen. While in the hospital pen, cows were milked separately in a conventional milking parlor consisting of one line with 8 milking units, until either full recovery or the end of the withdrawal period. Consequently, milking data from the hospital stay following diagnosis were unavailable and excluded from the analysis. If the cow failed to respond to 2 subsequent antibiotic treatments, the decision was made to perform abrupt dry-off of the affected quarter and return the cow to the AMS for regular milking, setting the milking units to not milk the affected quarter. After verifying the withdrawal times, cows subject to QDO were immediately moved out of the hospital pen. Nonetheless, as QDO was performed following clinical mastitis, the affected cows were housed in the hospital for variable time durations. As cows subject to QDO treatment returned to their original pens, their recovery was monitored using the milking system alert protocol, mainly based on milk yield deviations. However, cows were not subject to specific monitoring of the dried quarter, aside from the regular automatic health monitoring protocol in place on the farm.

To allow for milk yield follow-up, the inclusion criteria required that cows had milk yield records for the remaining quarters for at least 30 continuous days after QDO. In addition, due to the low prevalence of QDO in primiparous cows, only multiparous individuals were included in the study. Considering the herd average (SD) DIM at the peak of lactation [68 (49) d], cows were categorized based on their DIM at QDO into prepeak (≤68 DIM) and postpeak (>68 DIM) groups. In addition, cows were grouped based on their DQL as left front (**LF**), right front (**RF**), left rear (**LR**), and right rear (**RR**). Milk production data from one healthy control cow were matched to each affected cow based on DIM and parity number. Control cows were included using Excel (version 2510, Microsoft Corp.) by creating a column with the available matching cows' IDs and randomly selecting cows from this list (RAND function).

The statistical analyses were conducted using SAS 9.4 (SAS Institute Inc., Cary, NC) and completed separately for pre- and postpeak cows, as well as for quarter- and cow-level comparisons. Least squares means (SE) for daily average milk yield per functional quarter and per cow up to 30 d post QDO were calculated and compared among DQL groups (including matched control cows) using ANOVA for repeated measures analysis, using cow ID as the REPEATED statement (PROC MIXED). Compound symmetry was selected as the covariance structure, as we considered that the correlation between all pairs of repeated measures was similar between points and remained relatively constant across the comparison period. Initial univariable models using only DQL as the explanatory variable were followed by multivariable models that considered DIM at QDO and calving season as potential covariates. Milk yield curves following 30 d after QDO were built from the resulting daily milk yield LSM by DQL through repeated measures analysis.

Assumptions of constant variance and normality of residuals were evaluated using diagnostic plots. For all outcome variables, significant predictors were determined at *P*-value < 0.05; interaction terms and potential covariates remained in the models at *P*-value ≤ 0.10.

After excluding 11 cows that did not meet the inclusion criterion of having milk yield records for the remaining quarters for at least 30 continuous days after QDO, a total of 114 multiparous cows that calved between May 2022 and November 2024 and were subjected to QDO were included in the analysis. The distribution by lactation number was as follows: parity 2 (40.4%), parity 3 (33.3%), parity 4 (21.0%), and parity 5 (5.3%). Each affected cow was matched with one healthy control cow based on DIM and parity. Average (SD) DIM at QDO were 147.9 (99) d. The distribution of QDO in the prepeak group was 9 LF, 10 RF, 11 LR, and 10 RR. For the postpeak group, the distribution was 30 LF, 10 RF, 15 LR, and 19 RR.

Calving season was not significantly associated with any of the outcomes in analysis and therefore all the multivariable models only included DIM at QDO as covariate. The dynamics of the LSM for daily milk yield per individual quarter over the 30 d following QDO are shown by grouping cows according to their DQL and are presented separately for the prepeak (Figure 1) and postpeak (Figure 2) groups. In both figures the average milk yield from the front and rear quarters in control cows is included as a reference for comparison. These figures also present the total milk yield per cow during the same period, also including unaffected controls for comparison.

**Figure 1:**
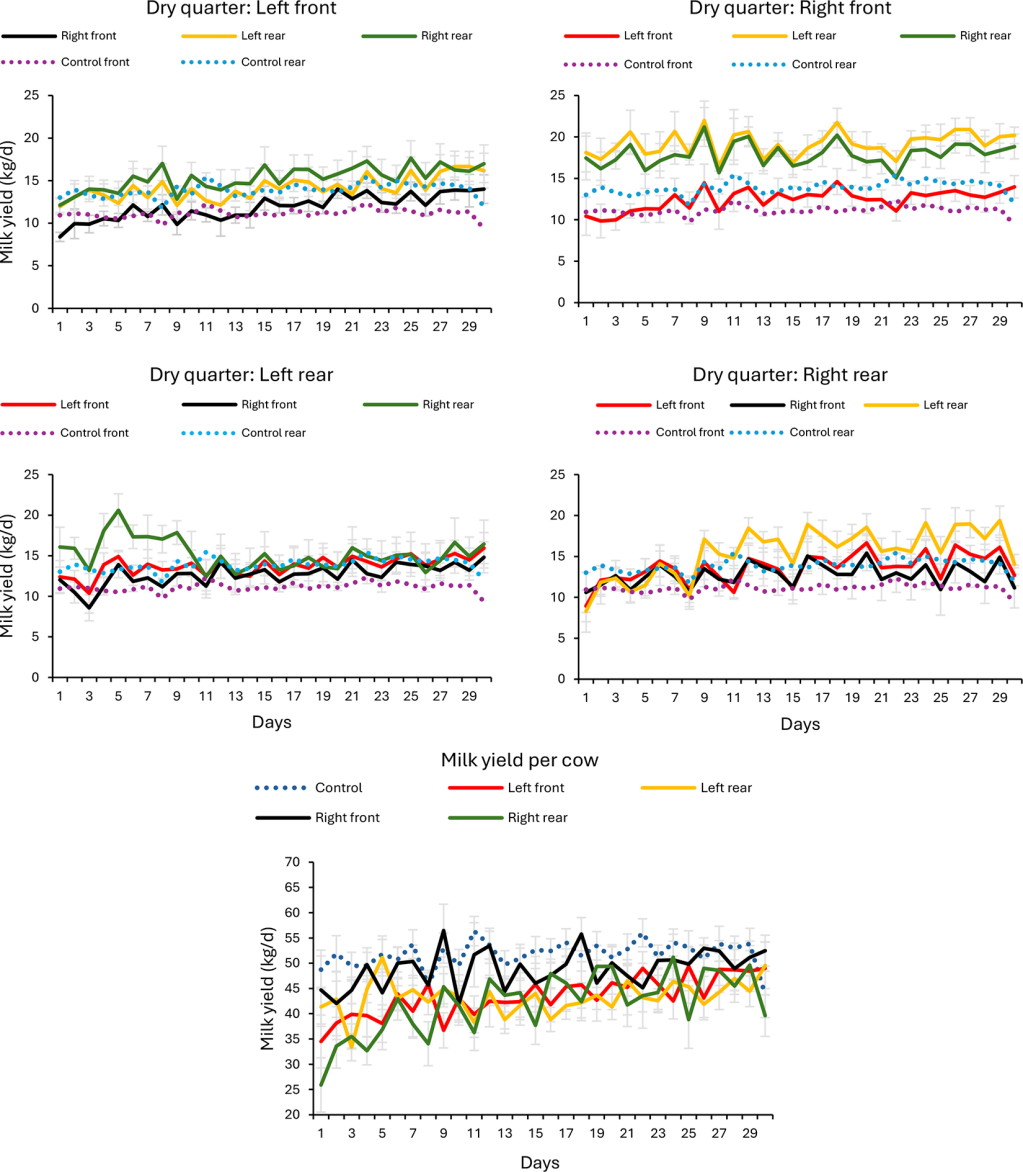
Daily milk yield per quarter (kg/d) in the 30 d following individual quarter dry-off before 69 DIM (average herd milk peak). Milk yield from the 3 remaining quarters is presented for cows with their left front (top left panel), right front (top right panel), left rear (middle left panel), or right rear quarter (middle right panel) dried off. The average milk yield from the front and rear quarters in control cows is included as a reference for comparison. The bottom panel presents average milk yield per cow, including unaffected controls (dotted blue line) matched by lactation number and DIM.

**Figure 2:**
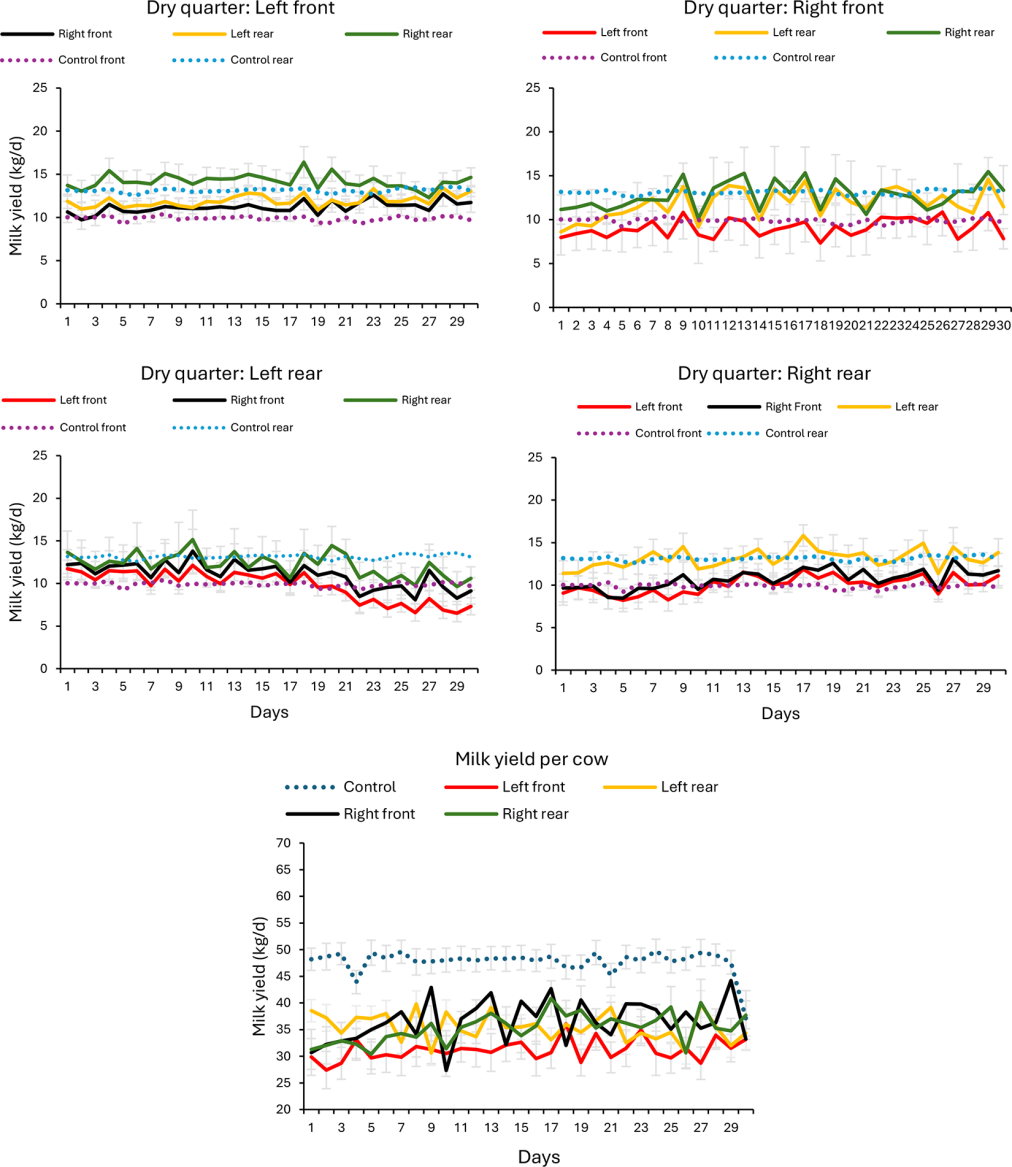
Daily milk yield per quarter (kg/d) in the 30 d following individual quarter dry-off after 68 DIM (average herd milk peak). Milk yield from the 3 remaining quarters is presented for cows with their left front (top left panel), right front (top right panel), left rear (middle left panel), or right rear quarter (middle right panel) dried off. The average milk yield from the front and rear quarters in control cows is included as a reference for comparison. The bottom panel presents average milk yield per cow, including unaffected controls (dotted blue line) matched by lactation number and DIM.

Average quarter milk yield per day for the 30 d following individual QDO are presented and compared in Table 1, grouping cows by DQL and separated by time of QDO as pre- or postpeak, including unaffected control cows for comparison. Total cow milk yields by group of DQL are also included in Table 1.

**Table 1:** Least squares means (SE) for milk yield (kg/d) at the quarter and at the cow level, classifying the cows by dry quarter location^1^

Item	Milk yield per functional quarter (kg/d)								Milk yield per cow (kg/d)	
	Left front		Right front		Left rear		Right rear			
Prepeak										
Dry quarter location (n)										
Left front (9)	—	—	11.6	(1.31)	14.7^ab^	(1.19)	15.6^ab^	(1.35)	43.0^ab^	(3.36)
Right front (10)	12.6	(0.86)	—	—	18.0^b^	(0.74)	17.4^b^	(1.00)	48.1^ab^	(1.74)
Left rear (11)	13.6	(1.30)	12.8	(1.13)	—	—	14.7^ab^	(1.81)	42.9^ab^	(3.19)
Right rear (10)	13.2	(1.05)	13.1	(1.43)	14.5^ab^	(1.78)	—	—	41.5^a^	(3.06)
Control (40)	11.0	(0.57)	11.0	(0.60)	13.8^a^	(0.82)	13.8^a^	(0.68)	51.7^b^	(1.85)
Postpeak										
Dry quarter location (n)										
Left front (30)	—	—	10.6	(0.44)	12.1	(0.87)	13.6	(0.78)	32.5^a^	(2.89)
Right front (10)	9.69	(1.24)	—	—	12.7	(1.23)	14.0	(2.00)	32.2^a^	(3.37)
Left rear (15)	11.6	(0.93)	12.5	(1.10)	—	—	12.8	(1.98)	35.5^a^	(2.35)
Right rear (19)	10.5	(1.08)	11.1	(1.32)	13.2	(0.97)	—	—	35.3^a^	(2.77)
Control (74)	9.26	(0.38)	9.71	(0.36)	12.9	(0.52)	12.7	(0.47)	47.1^b^	(1.70)

a,b Different superscripts within columns and pre- or postpeak categories indicate significant differences (*P* < 0.05).

1 The analyses were completed separately for pre- and postpeak cows. One healthy control cow was matched to each affected cow based on DIM and parity number as a reference for comparison. The model included DIM at quarter dry-off as covariate.

Differences in quarter milk yield were only identified for the prepeak group. Milk yields from the LR and RR quarters were smaller in control cows than in cows subjected to QDO of the RF quarter. When the total milk yield per cow was compared within the prepeak group, control cows had greater yield than cows with RR DQL. In the postpeak group, control cows had the highest milk yield compared with the 4 groups of cows with a dry quarter (Table 1).

Considering the findings of Hamann and Reichmuth (1990) and Skarbye et al. (2018), we hypothesized that following QDO, the remaining functional quarters would compensate for the loss of one quarter by increasing their milk yield, ultimately reaching levels comparable to those of unaffected control cows. A key limitation of our study was that QDO was performed following clinical mastitis, resulting in affected cows being housed in the hospital for variable time durations. This accommodation prevented consistent milk yield data collection during the baseline period previous to the QDO. Due to the absence of this critical pretreatment information, we opted for matching each affected cow with an unaffected control cow to allow for comparative analyses. Moreover, when comparing milk yield between QDO cows and healthy controls, certain additional impacts of mastitis, such as hospital transfers and potential systemic effects of the disease on affected cows, were not accounted for in the analysis. It should be also considered that only the cows that had milk yield records for the remaining quarters for at least 30 d after QDO were included in the study. In consequence, if a cow was sick and had to return to the hospital during this period, she was not considered in the study. This inclusion criterion could result in some degree of selection bias, as cows in suboptimal health status were not included in any calculation during this study. As the QDO procedure was completed by setting the milking units to not milking the affected quarter, there was a possibility that a dry quarter could be mistakenly milked. To address this issue, records were checked for no milk yield in the quarter submitted for dry-off during the whole study period.

Interestingly, as shown in Figure 1, Figure 2, milk yield trends over the subsequent 30 d post-dry-off were variable, depending on the remaining functional quarters. As anticipated based on the study from Skarbye et al. (2018), where early-lactation multiparous cows showed the greatest compensatory potential, quarters in cows categorized as prepeak (≤68 DIM) had greater milk yields and a more pronounced upward trend following QDO compared with those in the postpeak category (>68 DIM). Additionally, rear quarters produced more milk than front quarters across all groups.

The compensatory changes in quarter milk production after dry-off of one or more quarters has been investigated. In an early study by Hamann and Reichmuth (1990), the effects of leaving 1, 2, or 3 quarters per cow unmilked for 12 d were analyzed. Milk yield was recorded by quarter during the treatment period and for an additional 12 d after cows resumed normal milking. During the treatment phase, the average daily milk yield per cow declined to 26%, 59%, and 75% of the pretreatment baseline level when only 1, 2, or 3 quarters were milked, respectively. Nonetheless, daily milk yield increased in all quarters that were milked throughout the treatment period, with 14%, 10%, and 4% for cows milked in 1, 2, and 3 quarters, respectively.

Similar to the findings reported by Hamann and Reichmuth (1990), in our study, when quarter milk yields were compared between QDO cows and controls, the lowest numerical values per quarter were consistently established for control cows, except for the LR quarter in the postpeak group (Table 1). Nonetheless, these differences were only significant in the prepeak group, for milk yield in LR and the RR quarters when comparing control cows with cows with the RF quarter dried off. Interestingly, in a study by Skarbye et al. (2018), in agreement with the inconsistency of our results in pre- and postpeak cows, early-lactation multiparous cows subjected to QDO had the greatest compensatory potential.

Some limitations in the statistical analysis should be noted. The moderate sample size in our study may have contributed to the lack of significance in some comparisons. However, post hoc power calculations conducted in SAS (PROC POWER), based on observed differences in milk yield between cows subjected to QDO and control cows at both the quarter and cow level, indicated adequate statistical power (>80%). Conversely, if the assumption of constant covariance between any 2 repeated measurements was not fully met, some *P*-values may have been underestimated.

A possible explanation for the compensatory increase in milk production of the individual remaining quarters relates to changes in the blood flow and nutrient partitioning, with the consequent greater availability of nutrients per functional quarter in cows following QDO (Davis and Collier, 1985; Komatsu et al., 2005). This idea would align with the greater compensatory increases observed in the prepeak group, where potential cow negative energy balance would affect the rate of nutrient transport into the epithelial cells and result in a limiting factor in cows going through periods of greater milk yield (Grummer et al., 2004; Leduc et al., 2021). As reviewed by Gross (2022), in addition to substrate availability, the kinetics and hormonal regulation of transporters are influenced by the cow's physiological and hormonal stages (Zhao and Keating, 2007a,b; Davis et al., 2021) and may act as a limiting factor for milk synthesis during early lactation. It is also important to recognize that both the number and activity of milk-secreting cells fluctuate throughout lactation. As reported by Capuco et al. (2001), the number of secretory cells peaks at the onset of lactation, whereas milk yield per cell is initially low. This yield increases significantly from early to peak lactation and then stabilizes. The rise in milk production up to peak lactation is likely due to continued cellular differentiation rather than an increase in cell number. Conversely, the decline in milk yield after peak lactation is probably attributable to a reduction in the number of secretory cells rather than diminished cellular activity. Therefore, the mammary gland's capacity to respond to increased nutrient availability with higher milk output per quarter may be constrained during the later stages of lactation (Svennersten-Sjaunja and Olsson, 2005). Moreover, differences in the hormonal landscape during early and late lactation may also have an impact on the ability of the functional quarters to compensate for the absence of the dried quarter.

An interesting finding reported by Hamann and Reichmuth (1990) is that following temporary dry-off of one quarter for a period of 12 d, the increments in milk yield identified in the remaining milking continued above their pretreatment levels when milking was resumed in the adjacent quarters. This indicates that, in addition to changes associated with nutrient availability for milk synthesis, there is potential for more permanent changes associated with increments in the numbers of secretory cells.

It is important to note that, contrary to the compensatory effect of the individual remaining quarters, when total milk yield per cow was compared, control cows had greater milk yield than cows subjected to QDO. Whereas in the prepeak group significant differences were only observed between controls and cows with their RR quarter dry, in the postpeak category, control cows had greater milk yield than all the other 4 groups of cows with one QDO (Table 1).

The opposite direction in the differences in milk yield at the quarter or at the cow level indicates that the compensatory increase in milk production in each quarter post QDO is not enough to compensate for the lack of one quarter in the overall cow milk yield (Table 1). Notably, this inability of the affected cows to recover their yield is more evident during the postpeak period, when milk yield in control cows remained above the level of production of the 4 groups of QDO cows through the whole 30-d period (Table 1, Figure 2).

In agreement with these findings, in a study comparing QDO and continuous milking in subclinical mastitis cases, Skarbye et al. (2018) reported that the average production loss was 4.1 kg/d greater in cows subjected to QDO than in untreated controls. As in our study, the production loss depended on the DIM of the cow subjected to dry-off, with early-lactation cows showing the greatest compensatory potential. Supporting our findings on compensatory milk yield, Wieland and Skarbye (2024) reported a decrease in milk yield following QDO compared with their respective control groups. However, all QDO cows approached the yield of their control group by the third monthly test day.

In conclusion, following QDO, the levels of milk yield compensation in the remaining functional quarters were variable and smaller when QDO happened post milk yield peak. Nonetheless, in most cases overall milk yield remained lower in 3-quarter cows compared with unaffected controls.
